# Primary health care professionals’ practice in the face of leprosy: a scoping review

**DOI:** 10.1590/0034-7167-2023-0207

**Published:** 2024-06-14

**Authors:** Michelle Santos Macêdo, Nanielle Silva Barbosa, Priscilla Dantas Almeida, Júlia Oliveira Melo, Jonas Alves Cardoso, Telma Maria Evangelista de Araújo

**Affiliations:** I Universidade Federal do Piauí. Teresina, Piauí, Brazil; II Universidade Federal do Amazonas. Manaus, Amazonas, Brazil; III Faculdade Ciências Médicas de Minas Gerais. Belo Horizonte, Minas Gerais, Brazil; IV Universidade Federal do Rio Grande. Rio Grande, Rio Grande do Sul, Brazil

**Keywords:** Neglected Diseases, Leprosy, Health Personnel, Primary Health Care, Health Promotion, Enfermedades Desatendidas, Lepra, Personal de Salud, Atención Primaria de Salud, Promoción de la Salud

## Abstract

**Objectives:**

to identify Primary Health Care professionals’ practice in the face of leprosy.

**Methods:**

a scoping review, carried out between November 2022 and January 2023, conducted according to the methodological structure proposed by JBI and checklist Preferred Reporting Items for Systematic Reviews and Meta-Analyses extension for Scoping Reviews in six databases and additional literature.

**Results:**

the sample consisted of 11 articles, published between 2008 and 2022. The findings were synthesized into three categories: Early diagnosis and timely treatment; Physical disability prevention; and Household and social contact surveillance.

**Final Considerations:**

there is a need to align the practices carried out with those recommended by the Brazilian National Program for Leprosy Control and Elimination, as some were not identified in studies, which implies losses to qualified assistance directed to patient demands, with a view to control and elimination of leprosy.

## RESUMEN

**Objetivos:**

identificar las prácticas de los profesionales de la salud de Atención Primaria frente a la lepra.

**Métodos:**

revisión del alcance, realizada entre noviembre de 2022 y enero de 2023, realizada de acuerdo con el marco metodológico propuesto por JBI y *checklist Preferred Reporting Items for Systematic Reviews and Meta-Analyses extension for Scoping Reviews* en seis bases de datos y literatura adicional.

**Resultados:**

la muestra estuvo compuesta por 11 artículos, publicados entre 2008 y 2022. Los hallazgos se resumieron en tres categorías: Diagnóstico precoz y tratamiento oportuno; Prevención de discapacidades físicas; y Vigilancia de los contactos domésticos y sociales.

**Consideraciones Finales:**

es necesario alinear las prácticas realizadas con las recomendadas por el Programa Nacional para el Control y Eliminación de la Lepra, ya que algunas no fueron identificadas en los estudios, lo que implica pérdidas en la asistencia calificada y dirigida a las demandas del cliente, con una para el control y eliminación de la lepra.

## INTRODUCTION

Leprosy is a chronic infectious disease that, although curable, still remains endemic in several regions of the world, such as India, followed by Brazil and Indonesia. It is associated with unfavorable economic, social and environmental conditions. In Brazil, it is still considered an important public health problem, involving issues related to stigma, discrimination and social exclusion associated with the disease^(1, 2)^.

*Mycobacterium leprae* (M. *leprae*) is the etiological agent responsible for the disease, described in 1873 by the Norwegian doctor Gerhand Armauer Hansen, as an alcohol-acid-resistant bacillus that affects the skin and peripheral nerves, with the capacity to cause neural injuries, causing high disabling power. The main source of infection by the bacillus are untreated individuals affected by leprosy and with a high bacillary load who eliminate M. *leprae* through the upper airways^(3)^.

A The main strategy to achieve low endemic levels and reduce the burden of leprosy depends on the adoption of prevention and control measures, as these are essential activities to be carried out in health units as a way of promoting health and preventing diseases. In this regard, Primary Health Care (PHC) plays a fundamental role in controlling leprosy, acting as a gateway and organizer of care, in order to detect the disease, promote user access, reduce stigma and guarantee comprehensive care^(4)^.

Disease control actions must be developed in a decentralized and integrated manner by PHC professionals, mainly doctors and nurses, who must be able to: recognize the signs and symptoms of the disease early; correctly monitor therapeutic response and the side effects of uniform polychemotherapy (U-MDT) and anti-reaction medications; prevent and treat physical disabilities (PD); carry out health education with a focus on combating stigma; and examine household and social contacts^(5)^.

Considering that, to provide assistance to people with leprosy, PHC professionals must be qualified and have adequate knowledge about the disease, it is important to investigate the evidence on what practices these professionals adopt to combat this disease.

## OBJECTIVES

To identify PHC professionals’ practice in the face of leprosy.

## METHODS

### Study design

This is a scoping review, prepared according to the method proposed by JBI^(6)^. This method is recognized for its usefulness when it is intended to map and synthetize the scientific evidence that supports a given area of research as well as clarify definitions and identify gaps in knowledge, guiding the need for new investigations^(7, 8)^.

### Methodological procedure

A review protocol was developed that adopted the requirements proposed by the Preferred Reporting Items for Systematic Reviews and Meta-Analyses extension for Scoping Reviews (PRISMA-ScR)^(9)^, being registered in the Open Science Framework (OSF) (10.17605/OSF.IO/WCKMH).

To develop the study, the recommended steps were followed^(6)^, namely: determine the objectives and research question; define the inclusion and exclusion criteria; and define a search and selection strategy for evidence for extraction and compilation of results obtained, analysis and synthesis of evidence and presentation of results.

When constructing the research question, the PCC (Population, Concept and Context) strategy was used for a scoping review^(6)^. The following were defined: P: health professional; C: practices in relation to leprosy: and C: PHC. Based on these definitions, the guiding question was established: what practices are adopted by PHC health professionals in relation to leprosy?

Primary studies in Portuguese, English and Spanish, without temporal delimitation, were included. Letters to the editor, editorials, synthesis of event annals, expert opinions and articles that did not include the population, concept and context of interest of the study were excluded.

Additionally, there was a search for potentially eligible studies in articles’ bibliographic reference lists. Such mapping of literature occurred from the full reading of studies relevant to the scope of the topic under study.

### Data source and research strategy

The bibliographic survey period took place from November 2022 to January 2023. Initially, research was carried out in the following electronic databases: Latin American and Caribbean Literature in Health Sciences (LILACS); Medical Literature Analysis and Retrieval System Online (MEDLINE via PubMed); Cumulative Index to Nursing and Allied Health Literature (CINAHL via EBSCO); Web of Science; Scopus; and Embase. The terms used were selected from the Health Sciences Descriptors (DeCs), for databases in Portuguese, from the Medical Subject Headings (MeSH) and the CINAHL Subject Headings, a specific word used in the CINAHL database, for databases in English. The terms were combined with Boolean operators AND and OR, generating search expressions (Chart 1).

**Chart 1:** Terms, search expression used and number of studies in the databases

Database	Search expression	Number of studies identified
LILACS	((mh:(‘’*Pessoal de Saúde*”)) OR (‘’*Profissional de saúde*”) OR (‘’*Trabalhador da saúde*”)) AND ((mh:(*hanseniase*)) OR (*lepra*)) AND ((mh:(‘’*Atenção Primária à Saúde*”)) OR (‘’*Atenção Básica*”))	29
MEDLINE	((((“health personnel”[MeSH Terms]) OR (“healthcare workers”[All Fields])) OR (“healthcare worker”[All Fields])) AND ((“leprosy”[MeSH Terms]) OR (“hansen disease”[All Fields]))) AND ((“primary health care”[MeSH Terms]) OR (“primary care”[All Fields]))	21
CINAHL	((MH “Health Personnel”) OR “”Health Personnel”” OR “”Healthcare Workers”” OR “”Healthcare Worker””) AND ((MH “Leprosy”) OR “leprosy” OR “”hansen disease””) AND ((MH “Primary Health Care”) OR “”Primary Health Care”” OR “”Primary Care””)	6
Web of Science	(ALL=(‘’health personnel”) OR ALL=(‘’healthcare workers”) OR ALL=(‘’healthcare worker”)) AND (ALL=(Leprosy) OR ALL=(‘’hansen disease”)) AND (ALL=(‘’Primary Health Care”) OR ALL=(‘’Primary Care”))	50
Scopus	((TITLE-ABS-KEY (‘’health personnel”) OR TITLE-ABS-KEY (‘’healthcare workers”) OR TITLE-ABS-KEY (‘’healthcare worker”))) AND ((TITLE-ABS-KEY (leprosy) OR TITLE-ABS-KEY (‘’hansen AND disease”))) AND ((TITLE-ABS-KEY (‘’primary health care”) OR TITLE-ABS-KEY (‘’primary care”)))	32
Embase	(‘health care personnel’/exp OR ‘health care personnel’ OR ‘health care practitioner’) AND (‘leprosy’/exp OR ‘hansen disease’ OR ‘hansen’s disease’ OR ‘m. leprae infection’ OR ‘mycobacterium leprae infection’ OR ‘infection caused by m. leprae’ OR ‘infection caused by mycobacterium leprae’ OR ‘lepra’) AND (‘primary health care’/exp OR ‘primary health care’ OR ‘primary healthcare’)	54

Then, the articles found in the databases were exported to the Rayyan reference manager^(10)^, where titles and abstracts were analyzed, selected according to established inclusion criteria. Duplicates were considered only once. Subsequently, the lists of bibliographic references of selected studies were analyzed to identify other relevant studies.

The study selection process was carried out by two reviewers independently and simultaneously. Disagreements between reviewers regarding study inclusion, at any stage of development, were resolved through a third reviewer, after reading and analyzing the material in its entirety and deciding on the composition of the final sample.

### Data extraction

To extract data of interest from the studies, an instrument developed in consensus by the researchers was used, which included identification of authors, title, year, country of origin, journal, method, health professional and main practices mentioned. The process of extracting this information was carried out by two reviewers independently. In cases where divergences occurred, a third reviewer’s opinion was requested to reach a consensus.

Studies’ level of evidence was determined by the following classification proposed by JBI: level I – experimental studies; level II – quasi-experimental studies; level III – analytical observational studies; level IV – descriptive observational studies; level V – consensus and expert opinion^(11)^.

The main results of included studies were analyzed in a qualitative and descriptive way, according to their content^(6, 7, 8, 9, 10, 11)^, and presented in the form of charts.

## RESULTS

**Figure 1:**
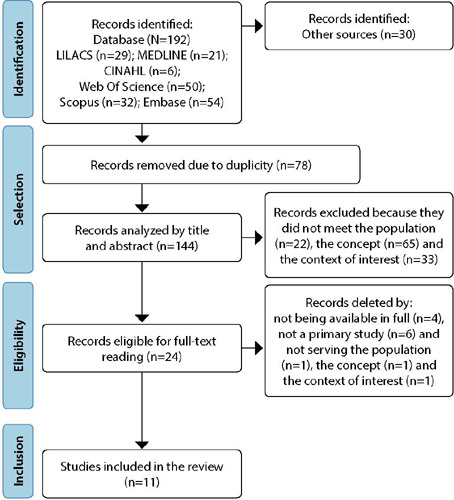
Study selection and inclusion process flowchart (PRISMA-ScR, 2018)

The evidence survey initially identified 222 studies, 192 in databases and 30 in reference lists, of which 78 were excluded due to duplication. Of the 144 records selected for title and abstract reading, 24 were eligible for full reading, but four were not retrieved in the literature. Finally, 11 articles were included in this review (Figure 1).

The period of publication of the findings varied between the years 2008 and 2022, and no time frame was defined. All studies were developed and published in Brazil. All selected studies presented level of evidence IV, characterizing themselves as descriptive observational studies. The articles were analyzed in full, and those that corresponded to the objectives and research question of this review were extracted (Chart 2).

**Chart 2:** Description of articles included in the scoping review according to author, title, year of publication, country, journal, method, health professional, main practices reported and level of evidence

Authors/title	Year/country/journal	Method/health professional	Main practices mentioned	Level of evidence
Ribeiro MDA, Castillo IDS, Silva JCA, Oliveira SB "*A visão do profissional enfermeiro sobre o tratamento da hanseníase na atenção básica*”^(12)^	2017/Brazil/*Revista Brasileira em Promoção da Saúde*	Qualitative descriptive study, with nine nurses	Nursing consultation; absence of active search de cases; late diagnosis; supervised treatment; adverse reactions to medication; management of leprosy reactions; dermatoneurological assessment and degree of PD; contact surveillance; combating stigma; health education.	IV
Pinheiro JDJG, Gomes SCS, Aquino DMCD, Caldas ADJM “*Aptidões cognitivas e atitudinais do enfermeiro da atenção básica no controle da hanseníase*”^(13)^	2017/Brasil/*Revista Baiana de Enfermagem*	Descriptive study, with 101 nurses	Nursing consultation; absence of active search de cases; late diagnosis; deficit of information about supervised treatment; absence of contact surveillance; health education.	IV
Pereira AJ, Helene LMF, Pedrazini ES, Martins CL, Vieira CSDCA “*Atenção básica de saúde e a assistência em hanseníase em serviços de saúde de um município do Estado de São Paulo*”^(14)^	2008/Brazil/*Revista Brasileira de Enfermagem*	Descriptive-exploratory study with an epidemiological basis, with ten health professionals (nurses, doctors, nursing assistants, social worker, psychologist and municipal health secretary)	Nursing consultation; absence of active search de cases; early diagnosis; reporting; supervised treatment; dermatoneurological assessment and degree of PD; contact surveillance; guidance on BCG; combating stigma; psychological, ophthalmological, dental and physiotherapy assessment; home visit; health education.	IV
Vieira NF, Lanza FM, Lana FCF, Martinez-Riera JR “*Avaliação dos atributos da atenção primária à saúde nas ações de controle da hanseníase*”^(15)^	2018/Brazil/*Revista Enfermagem UERJ*	Evaluative and cross-sectional study, involving 251 primary care professionals (doctor, nurse, community health worker and managers)	Absence of active search for cases; late diagnosis; supervised treatment; lack of assessment of PD degree; absence of contact surveillance; absence of health education.	IV
Costa IMD, Morais ATND, Nascimento RGD, Lima JPM, Santos MEDMAD, Dias GADS, et al “*Conhecimento do fisioterapeuta da atenção primária à saúde sobre a atuação profissional em pacientes com hanseníase*”^(16)^	2022/Brazil/*Revista* *Eletrônica* *Acervo Saúde*	Descriptive, cross-sectional, exploratory and qualitative study, with nine physiotherapists	Absence of active search de cases; late diagnosis; incomplete simplified neurological assessment; health education; inadequate guidance on self-care.	IV
Oliveira CMD,Linhares MSC, Neto FRGX, Mendes IMVP, Kerr LRFS “*Conhecimento e práticas dos agentes comunitários de saúde sobre hanseníase em um município hiperendêmico*”^(17)^	2018/Brazil/*Saúde em Revista*	Exploratory-descriptive study, using a quantitative and qualitative approach, with 51 community health workers	Knowledge about the clinical signs and symptoms of leprosy; active search for cases; supervised treatment; adverse reactions to medication; contact surveillance; guidance on BCG; guidance on self-care; home visit; combating stigma; health education.	IV
Lanza FM, Lana FCF “*O processo de trabalho em hanseníase: tecnologias e atuação da equipe de saúde da família*”^(18)^	2011/Brazil/*Texto & Contexto Enfermagem*	Qualitative research, with 45 health professionals (doctor, nurse, community health worker and managers)	Nursing consultation; active search for cases; late diagnosis; supervised treatment; adverse reactions to medication; management of leprosy reactions; dermatoneurological assessment and degree of PD and contact surveillance; home visit; health education.	IV
Rodrigues FF, Calou CGP, Leandro TA, Antezana FJ, Pinheiro AKB, Silva VMD, et al “*Conhecimento e prática dos enfermeiros sobre hanseníase: ações de controle e eliminação*”^(19)^	2015/Brazil/*Revista Brasileira de Enfermagem*	Evaluative and qualitative study, with 16 nurses	Nursing consultation; active search for new cases; early diagnosis; supervised treatment; management of leprosy reactions; dermatoneurological assessment and degree of PD; contact surveillance; guidance on BCG; combating stigma; guidance on self-care; home visit; health education.	IV
Carvalho APM, Fabri ACOC, Lanza FM, Lopes FN, Lana FCF “*Integração das ações de controle da hanseníase sob a perspectiva dos profissionais da saúde*”^(20)^	2015/Brazil/*Revista Enfermagem UFPE*	Qualitative study, with 54 health managers and professionals (doctor, nurse, nursing technician, nursing assistant and community health worker)	Absence of active search; late diagnosis; supervised treatment; adverse reactions to medication; lack of assessment of PD degree; contact surveillance; home visit; absence of health education.	IV
Sousa GS, Silva RLF, Xavier MB “*Atributos da atenção primária em saúde no controle da hanseníase: ótica do enfermeiro*”^(21)^	2017/Brazil/*Revista Baiana Enfermagem*	Evaluatve study, with 11 nurses	Nursing consultation; absence of active search; early diagnosis; lack of dermatoneurological assessment and degree of PD; contact surveillance; family counseling; health education.	IV
Oliveira AG, Camargo CG “*Hanseníase: conhecimentos teóricos e práticos de profissionais de enfermagem que atuam na atenção básica*”^(22)^	2020/Brazil/*Salusvita*	Observational, cross-sectional and analytical study, with 42 nurses	Nursing consultation; absence of active search; late diagnosis; deficiency in dermatoneurological assessment and degree of PD; deficit of information about supervised treatment.	IV

*BCG - Bacillus Calmette-Guérin; PD – physical disability.*

Study content was analyzed, and the themes identified allowed them to be grouped according to similarity of information, creating three thematic categories: Early diagnosis and timely treatment; Physical disability prevention; and Household and social contact surveillance.

## DISCUSSION

It is of fundamental importance that health professionals, faced with suspected or confirmed cases of leprosy, carry out their practice in accordance with actions recommended by the Brazilian National Program for Leprosy Control and Elimination (PNCEH). The adoption of these actions and strategies, based on a theoretical-practical framework recommended by health authorities, allows for adequate case management, early diagnosis, access to timely treatment, PD prevention and household contact surveillance, which guarantees the recovery of a person affected by the disease^(23)^.

From this perspective, these actions recommended by the PNCEH were used to guide the process of discussing the findings identified in the studies.

### Early diagnosis and timely treatment

The multidisciplinary health team is essential human capital for valuing health care practices in the community, performing complex work with an emphasis on articulating and developing comprehensive user care^(24)^. Nurses, as one of the professionals on the team, become a key element in carrying out the actions proposed by the PNCEH, providing comprehensive care to patients, from diagnosis to completion of treatment^(25)^.

Nursing consultation is extremely important in leprosy control actions, as, in addition to creating trust and bonding with patients, it provides information and clarification about the disease to understand clinical manifestations, the need for adherence to treatment, communicator control, priority of cure and disability prevention. Therefore, nurses must act on the individual needs of each patient and provide quality care^(12, 13, 14, 18, 19, 21, 22)^.

The search for suspected leprosy patients can be carried out actively and passively. Active search is part of the PHC team’s work through the screening of users through campaigns, collective examination and community mobilization^(18, 19)^. It is a fundamental strategy for discovering cases of the disease which, when undervalued, contributes to expanding the margins of epidemiological silence^(12, 13, 14, 15, 16, 20, 21, 22)^. Passive search occurs when cases seek the health unit via spontaneous demand or referral, presenting dermatological complaints^(26)^. These strategies, especially the first, contribute to timely diagnosis, essential for preventing disabilities and/or worsening of cases.

Leprosy diagnosis is clinical-epidemiological, carried out through a physical examination of patients, in which thickened peripheral nerves and/or skin lesions or areas of skin with changes in thermal and/or painful and/or tactile sensitivity are observed and autonomic changes^(27)^. Early diagnosis and appropriate treatment are essential conditions to interrupt transmission and reduce the physical and social consequences of the disease. There is an emphasis on diagnosis in three studies^(14, 19, 21)^. Late diagnosis appears to be associated with a lack of professional training and delays in starting treatment, which contributes to greater neural damage to patients and worsening of the disease^(12, 13, 15, 16, 18, 20, 21, 22)^. The absence of the practice of early diagnosis in children under 15 years of age stands out, which contributes to relevant rates of endemicity^(19)^.

After diagnosis, treatment begins through U-MDT administration, a treatment recommended by the World Health Organization (WHO) from July 1, 2021. Treatment is based on a combination of three medications (rifampicin, dapsone and clofazimine) for a period of six months for paucibacillary clinical forms and 12 months for multibacillary^(28)^. The medications that make up UMDT cause uncomfortable adverse effects, being one of the main reasons for interrupting or abandoning treatment^(29)^. Among the studies, the lack of information among professionals about the appropriate treatment of leprosy stood out^(13, 14, 15, 16, 17, 18, 19, 20, 21, 22)^.

U-MDT has supervised doses, considered when patients attend the health service every 28 days to receive rifampicin, dapsone and clofazimine. The absence of patients to receive the dose means continued transmission of the disease and resistance of the bacillus to the medication^(28)^. Therefore, supervised treatment contributes to reducing treatment abandonment and increasing the number of people cured^(12, 14, 15, 17, 18, 19, 20)^.

Adherence to treatment is one of the greatest and most complex challenges for the health care system. The home visit is important to build the bond between professional and patient^(30)^ to establish and agree on the responsibilities inherent to each of these actors during treatment and enable comprehensive attention to patient demands with the aim of strengthening this relationship and developing a targeted care plan, considering that leprosy treatment requires a prolonged period^(14, 17, 18, 19, 20)^. At this time, the importance of adherence to treatment should be reinforced, encouraging self-care, providing guidance to patient and family and assessing contacts with the aim of interrupting the chain of transmission^(31)^.

During treatment, clinical complications, adverse reactions to treatment^(^12,17-18,20^)^, leprosy reactions, doubts regarding diagnosis and management may appear. These cases impose the need for referrals to reference services^(32)^. Leprosy reactions are phenomena of increased disease activity, with clinical worsening that can occur before, during or after the end of treatment with U-MDT. These reactions result from acute inflammation caused by the host’s immune system attacking the bacillus^(12, 18, 19)^. It is recommended that patients undergo a dental examination, as infectious foci can trigger the onset of reactional episodes^(33)^.

In order to provide complete assistance, the importance of interdisciplinary collaboration, effective communication and continuous commitment between the different professionals who make up health care teams stands out, including nurses, dermatologists, ophthalmologists, psychologists and occupational therapists, with the aim of promoting comprehensive and quality care for patients with leprosy^(12, 14)^.

### Physical disability prevention

One of the focuses of the Global Leprosy Strategy 2021-2030, launched by the WHO in 2021, is PD, with recommendations for strategic operational modifications. Among the new goals are zero leprosy, zero infection and disease and zero disability by 2030. This initiative occurs in response to the growing number of cases with degree 2 PD that has been occurring in the opposite direction of reducing the global detection of the disease^(34)^.

Patients’ Degree of Physical Disability (GIF) must be assessed at the time of diagnosis as well as upon discharge due to cure. However, this action, which is fundamental in the management of leprosy, is often neglected. Among the studies analyzed, four addressed this practice^(12, 14, 18, 19)^. GIF assessment is an epidemiological indicator that determines the early diagnosis and the success of activities aimed at interrupting the transmission chain^(28)^.

GIF assessment also makes it possible to plan actions to prevent disabilities and measure the quality of health care^(35)^. It is important that all health professionals are properly trained to carry out GIF assessment in patients with leprosy. However, the research results point to a worrying gap in this competence among professionals who work in PHC. In the analyzed context, deficiencies were identified with regard to simplified neurological assessment (SNA) and the ability to perform muscle strength and sensitivity tests of the eyes, hands and feet, which suggests the need for better preparation of these professionals^(13, 15, 16, 20, 21, 22)^.

SNA is recommended by the Ministry of Health as a means of assessing the impairments arising from nerve injuries caused by M. *leprae.* GIF assessment is a measure that indicates the existence of loss of protective sensitivity and/or visible deformity as a result of neural injury, directing professionals towards disability prevention and rehabilitation practices. This assessment identifies sensorimotor deficiencies in the eyes, hands and feet, and classifies them into GIF 0, 1 and 2. Degree 0 characterizes patients who do not manifest any problems caused by leprosy in their hands, feet and eyes; degree 1 occurs when PD is not detectable by inspection or visual acuity testing, but there is a decrease in protective sensitivity or a reduction in muscle strength in the hands, feet and/or eyes; and degree 2 is determined by the presence of visible physical disabilities or loss of visual acuity caused by leprosy neuropathy^(1)^.

The bacteria’s tropism for peripheral nerves is responsible for the disabling potential of the disease, which can generate deformities and disabilities, especially in the eyes, hands and feet. It is extremely important to highlight that leprosy treatment and management require an interdisciplinary approach, in addition to active collaboration between health professionals and patients, through early diagnosis and monitoring from the beginning until discharge, to prevent or minimize PD that cause limitations and promote prejudice and social exclusion in those affected^(36)^.

### Household and social contact surveillance

Household contact surveillance is another important action in controlling leprosy in PHC through active case detection, considering that they represent a population at greater risk of becoming ill due to greater exposure to the bacillus. In the anamnesis regarding the signs and symptoms of the disease, a dermatoneurological examination of all contacts of new cases, regardless of operational classification, immunoprophylaxis with the Bacillus Calmette-Guérin (BCG) vaccine, as well as follow-up for a period of at least five years after the diagnosis of the reported case^(28)^.

Contact surveillance aims to discover new cases among those who live or have lived for a long time with a newly diagnosed case of leprosy^(12, 14, 17, 18, 19, 20, 21)^, in addition to discovering possible sources of infection at home (family) or outside it (social)^(37)^. Challenges to leprosy control include continued transmission of the bacillus, difficulties in monitoring contacts and limited knowledge about transmission. Leprosy prevention is based on the detection of patients’ contacts^(38)^, especially family contacts, as this is the main determinant for the maintenance of incidence levels^(20)^. Dermatoneurological assessment is used to assess the presence of signs of leprosy in contact.

Dermatoneurological assessment, a key element in leprosy diagnosis, has not been carried out as frequently as expected in contact assessment. It must be carried out annually, for five years, regardless of whether they are family members or not, as they are the most vulnerable to developing the disease^(39)^. This practice, when not carried out, contributes to the spread of the bacillus and the development of PD, as many professionals do not feel safe or qualified enough to carry it out in their contacts^(15, 16, 20, 21)^. In the first assessment, if the contact does not show signs of leprosy, they should be assessed regarding their BCG vaccination status and taken to the vaccination room if necessary.

The BCG vaccine must be administered to contacts without signs and symptoms of the disease at the time of dermatoneurological assessment^(14, 17, 18, 19)^. Vaccine administration depends on vaccination history and/or presence of a vaccine scar, and follows the following guidelines: absence of BCG vaccine scar -one dose; a vaccine scar – one dose; two vaccination scars – do not vaccinate^(37)^. In most of the studies reviewed^(12, 13, 15, 16, 18, 20, 21, 22)^, no guidance and referral of contacts for BCG was observed. The lack of guidance at the time of consultations can be minimized with the practice of popular health education which, developed by all health care protagonists, becomes a tool of great impact.

Health education in relation to leprosy is a tool for interacting knowledge practices involved in the health-disease process^(12, 14, 17, 18, 19, 21)^ capable of enhancing processes that imply early diagnosis, awareness, prevention of transmission, adherence to treatment, mental health promotion, disability prevention and community support. Nursing plays a central role in the training process for prevention and promotion of care for this patient^(40)^. This practice, when not carried out due to professionals’ lack of knowledge regarding the disease, can contribute to late diagnosis and non-adherence to treatment^(13, 15, 16, 20, 22)^.

### Study limitations

One of the limitations identified is related to the fact that the review was only processed with national productions, which limits the comparison with the reality of other countries and regions of the world.

### Contributions to health

Identifying evidence made it possible to present a synthesis of the practices adopted, contributing to the dissemination of knowledge among the community, professionals and health services, with the aim of improving care related to the control of leprosy cases.

## FINAL CONSIDERATIONS

The study made it possible to identify the main practices adopted by PHC professionals in relation to leprosy, such as management of leprosy reactions, assessment of contacts, combating stigma, health education, supervised treatment, home visit, guidance on self-care, search for new cases, among others. However, a finding that points to the neglect of priority actions to control leprosy, such as early diagnosis, carrying out SNA and activities related to household and social contact surveillance, has become worrying.

Thus, there is a need to align the practices carried out with those recommended by the PNCEH, considering that some were not identified in the reviewed studies. In PHC, it is of great importance to implement initiatives aimed at health professionals’ continuing education with the purpose of strengthening their technical training, aiming to improve the effectiveness in controlling and eliminating leprosy as a public health problem.
