# Supplementary material for: Primary health care professionals’ practice in the face of leprosy: a scoping review
Source: Rev Bras Enferm. 2024 Jun 14;77(2):e20230207. doi: 10.1590/0034-7167-2023-0207 (PMC11178311; doi:10.1590/0034-7167-2023-0207)
Supplement: Translation

# Translation: Práticas dos profissionais de saúde da atenção primária diante da hanseníase: revisão de escopo

**DOI:** 10.1590/0034-7167-2023-0207pt

**Authors:** Michelle Santos Macêdo, Nanielle Silva Barbosa, Priscilla Dantas Almeida, Júlia Oliveira Melo, Jonas Alves Cardoso, Telma Maria Evangelista de Araújo

**Affiliations:** I Universidade Federal do Piauí. Teresina, Piauí, Brasil; II Universidade Federal do Amazonas. Manaus, Amazonas, Brasil; III Faculdade Ciências Médicas de Minas Gerais. Belo Horizonte, Minas Gerais, Brasil; IV Universidade Federal do Rio Grande. Rio Grande, Rio Grande do Sul, Brasil

**Keywords:** Doenças Negligenciadas, Hanseníase, Pessoal de Saúde, Atenção Primária à Saúde, Promoção da Saúde

## Abstract

**Objetivos:**

identificar as práticas dos profissionais de saúde da Atenção Primária diante da hanseníase.

**Métodos:**

revisão de escopo, realizada entre novembro de 2022 e janeiro de 2023, conduzida de acordo com a estrutura metodológica proposta pelo JBI e *checklist Preferred Reporting Items for Systematic Reviews and Meta-Analyses extension for Scoping Reviews* em seis bases de dados e literatura adicional.

**Resultados:**

a amostra foi composta por 11 artigos, publicados entre 2008 e 2022. Os achados foram sintetizados em três categorias: Diagnóstico precoce e tratamento oportuno; Prevenção das incapacidades físicas; e Vigilância dos contatos domiciliares e sociais.

**Considerações Finais:**

há a necessidade de alinhamento das práticas realizadas com o preconizado pelo Programa Nacional de Controle e Eliminação da Hanseníase, pois algumas não foram identificadas nos estudos, o que implica prejuízos à assistência qualificada e direcionada às demandas do cliente, com vistas ao controle e eliminação da hanseníase.

## INTRODUÇÃO

A hanseníase é uma doença infecciosa de evolução crônica que, embora curável, ainda permanece endêmica em várias regiões do mundo, como Índia, seguido do Brasil e Indonésia. Está associada a condições econômicas, sociais e ambientais desfavoráveis. No Brasil, ainda é considerada um importante problema de saúde pública, envolvendo questões relacionadas ao estigma, discriminação e exclusão social associadas à doença^(1, 2)^.

O *Mycobacterium leprae* (M. *leprae*) é o agente etiológico responsável pela doença, descrito em 1873, pelo médico norueguês Gerhand Armauer Hansen, como um bacilo álcool-ácido resistente que atinge a pele e nervos periféricos, com capacidade de ocasionar lesões neurais, conferindo alto poder incapacitante. A principal fonte de infecção pelo bacilo são indivíduos acometidos pela hanseníase não tratados e com alta carga bacilar que eliminam o M. *leprae* pelas vias aéreas superiores^(3)^.

A principal estratégia para atingir baixos níveis endêmicos e redução da carga da hanseníase depende da adoção de medidas de prevenção e controle, pois são atividades essenciais a serem realizadas nas unidades de saúde como forma de promoção da saúde e prevenção de agravos. Nesse sentido, a Atenção Primária à Saúde (APS) possui papel fundamental no controle da hanseníase, atuando como porta de entrada e ordenadora do cuidado, a fim de detectar a doença, favorecer o acesso ao usuário, reduzir o estigma e garantir a integralidade da assistência^(4)^.

As ações de controle da doença devem ser desenvolvidas de forma descentralizada e integrada pelos profissionais da APS, principalmente por médicos e enfermeiros, que devem estar aptos a: reconhecer precocemente os sinais e sintomas da doença; acompanhar corretamente a resposta terapêutica e os efeitos colaterais da poliquimioterapia única (PQT-U) e dos medicamentos antirreacionais; prevenir e tratar as incapacidades físicas (IF); realizar educação em saúde com foco no combate ao estigma; e examinar contatos domiciliares e sociais^(5)^.

Considerando-se que, para realização da assistência às pessoas com hanseníase, os profissionais da APS devem estar qualificados, possuindo conhecimentos adequados sobre a doença, torna-se relevante investigar as evidências sobre quais práticas esses profissionais adotam para atuar no enfrentamento dessa enfermidade.

## OBJETIVOS

Identificar as práticas dos profissionais de saúde da APS diante da hanseníase.

## MÉTODOS

### Tipo de estudo

Trata-se de estudo de revisão de escopo (*scoping review*), elaborado conforme o método proposto pelo JBI^(6)^. Esse método possui utilidade reconhecida quando se pretende mapear e resumir as evidências científicas que sustentam uma determinada área de pesquisa, bem como esclarecer as definições e identificar lacunas do conhecimento, orientando a necessidade de novas investigações^(7, 8)^.

### Procedimento metodológico

Foi elaborado um protocolo de revisão que adotou os requisitos propostos pelo *Preferred Reporting Items for Systematic Reviews and Meta-Analyses extension for Scoping Reviews* (PRISMA-ScR)^(9)^, sendo registrado na plataforma *Open Science Framework* (OSF) (10.17605/OSF.IO/WCKMH).

Para o desenvolvimento do estudo, seguiram-se as etapas recomendadas^(6)^, nomeadamente: determinar os objetivos e questão de pesquisa; delimitar os critérios de inclusão e exclusão; e definir estratégia de busca e seleção das evidências para extração e compilação dos resultados obtidos, análise e resumo de evidência e apresentação dos resultados.

Na construção da pergunta de pesquisa, utilizou-se a estratégia PCC (População, Conceito e Contexto) para uma *scoping review* ^(6)^. Foram definidos: P: profissional de saúde; C: práticas em relação à hanseníase: e C: APS. Com base nessas definições, foi estabelecida a pergunta norteadora: quais as práticas adotadas pelos profissionais de saúde da APS em relação à hanseníase?

Foram incluídos na revisão estudos primários em português, inglês e espanhol, sem delimitação temporal. Foram excluídos carta ao editor, editoriais, resumos de anais de eventos, opinião de especialistas e artigos que não contemplavam a população, o conceito e o contexto de interesse do estudo.

De forma adicional, houve busca de estudos potencialmente elegíveis nas listas de referências bibliográficas dos artigos. Tal mapeamento da literatura ocorreu a partir da leitura na íntegra dos estudos relevantes para o escopo da temática em estudo.

### Fonte dos dados e estratégia de pesquisa

O período de levantamento bibliográfico ocorreu durante os meses de novembro de 2022 a janeiro de 2023. Inicialmente, realizou-se a pesquisa nas seguintes bases eletrônicas de dados: Literatura Latino-Americana e do Caribe em Ciências da Saúde (LILACS); *Medical Literature Analysis and Retrievel System Online* (MEDLINE via PubMed); *Cumulative Index to Nursing and Allied Health Literature* (CINAHL via EBSCO); *Web of Science*; Scopus; e Embase. Os termos utilizados foram selecionados a partir dos Descritores em Ciências da Saúde (DeCs), para as bases na língua portuguesa, dos *Medical Subject Headings* (MeSH) e do CINAHL *Subject Headings*, vocábulo específico utilizado na base CINAHL, para as bases na língua inglesa. Os termos foram combinados com os operadores booleanos *AND* e *OR*, gerando expressões de busca (Quadro 1).

**Quadro 1:** Termos, expressão de busca utilizadas e quantitativo de estudos nas bases de dados

Base de dados	Expressão de busca	Quantitativo de estudos identificados
LILACS	((mh:(‘’Pessoal de Saúde”)) *OR* (‘’Profissional de saúde”) *OR* (‘’Trabalhador da saúde”)) *AND* ((mh:(hanseniase)) *OR* (lepra)) *AND* ((mh:(‘’Atenção Primária à Saúde”)) *OR* (‘’Atenção Básica”))	29
MEDLINE	((((“*health personnel*”[MeSH Terms]) *OR* (“*healthcare workers*”[*All Fields*])) *OR* (“*healthcare worker*”[*All Fields*])) *AND* ((“*leprosy*”[MeSH *Terms*]) *OR* (“*hansen disease*”[*All Fields*]))) *AND* ((“*primary health care*”[MeSH *Terms*]) *OR* (“*primary care*”[*All Fields*]))	21
CINAHL	((MH “*Health Personnel*”) *OR* “”*Health Personnel*”” *OR* “”*Healthcare Workers*”” *OR* “”*Healthcare Worker*””) *AND* ((MH “*Leprosy*”) *OR* “*leprosy*” *OR* “”*hansen disease*””) *AND* ((MH “*Primary Health Care*”) *OR* “”*Primary Health Care* ”” *OR* “”*Primary Care*””)	6
*Web of Science*	(*ALL*=(‘’*health personnel*”) *OR ALL*=(‘’*healthcare workers*”) *OR ALL*=(‘’*healthcare worker*”)) *AND* (*ALL*=(*Leprosy*) *OR ALL*=(‘’*hansen disease*”)) *AND* (*ALL*=(‘’*Primary Health Care*”) *OR ALL*=(‘’*Primary Care*”))	50
Scopus	((*TITLE-ABS-KEY* (‘’*health personnel*”) *OR TITLE-ABS-KEY* (‘’*healthcare workers*”) *OR TITLE-ABS*- *KEY* (‘’*healthcare worker*”))) *AND* ((*TITLE-ABS-KEY* (*leprosy*) *OR TITLE-ABS-KEY* (‘’*hansen AND disease*”))) *AND* ((*TITLE-ABS-KEY* (‘’*primary health care*”) *OR TITLE-ABS-KEY* (‘’*primary care*”)))	32
Embase	(*‘health care personnel’*/exp *OR ‘health care personnel’ OR ‘health care practitioner’*) *AND* (*‘leprosy’*/*exp OR ‘hansen disease’ OR ‘hansen’s disease’ OR ‘m. leprae infection’ OR ‘mycobacterium leprae infection’ OR ‘infection caused by* m*. leprae’ OR ‘infection caused by mycobacterium leprae’ OR ‘lepra’*) *AND* (*‘primary health care’*/*exp OR ‘primary health care’ OR ‘primary healthcare’*)	54

Em seguida, os artigos encontrados nas bases foram exportados para o gerenciador de referências *Rayyan* ^(10)^, onde foram analisados títulos e resumos, selecionados conforme critérios de inclusão estabelecidos. Produções duplicadas foram consideradas uma única vez. Posteriormente, as listas das referências bibliográficas das produções selecionadas foram analisadas para a identificação dos demais estudos relevantes.

O processo de seleção dos estudos foi realizado por dois revisores, de forma independente e simultânea. As divergências entre os revisores quanto à inclusão do estudo, em qualquer etapa do desenvolvimento, resolveram-se por intermédio de um terceiro revisor, após a leitura e análise do material na íntegra e desempate na composição da amostra final.

### Extração de dados

Para a extração de dados de interesse dos estudos, foi utilizado instrumento elaborado em consenso pelos investigadores, que incluía identificação dos autores, título, ano, país de origem, periódico, método, profissional de saúde e principais práticas referidas. O processo de extração dessas informações foi realizado por dois revisores, de forma independente. Nos casos em que ocorreram divergências, foi solicitado parecer de um terceiro revisor para se atingir um consenso.

O nível de evidência das produções foi determinado pela seguinte classificação proposta pelo JBI: nível I – estudos experimentais; nível II – estudos quase-experimental; nível III – estudos observacionais analíticos; nível IV – estudos observacionais descritivos; nível V – consenso e opinião de especialistas^(11)^.

Os principais resultados dos estudos incluídos foram analisados de maneira qualitativa e descritiva, conforme seu conteúdo^(^6-11^)^, e apresentados em forma de quadros.

## RESULTADOS

O levantamento das evidências identificou inicialmente 222 produções, sendo 192 nas bases de dados e 30 das listas de referência, das quais 78 foram excluídas por duplicidade. Dos 144 registros selecionados para leitura de título e resumo, 24 foram elegíveis para a leitura na íntegra, porém quatro não foram recuperados na literatura. Por fim, 11 artigos foram incluídos nesta revisão (Figura 1).

**Figura 1:** Fluxograma do processo de seleção e inclusão do estudo (PRISMA-ScR, 2018)

O período de publicação dos achados variou entre os anos de 2008 e 2022, e não foi delimitado marco temporal. Todas as produções foram desenvolvidas e publicadas no Brasil. Todos os estudos selecionados apresentaram nível de evidência IV, caracterizando-se como estudos observacionais descritivos. Os artigos foram analisados na íntegra, sendo extraídos aqueles que correspondiam aos objetivos e à pergunta de pesquisa desta revisão (Quadro 2).

**Quadro 2:** Descrição dos artigos incluídos na revisão de escopo segundo autor, título, ano de publicação, país, periódico, método, profissional de saúde, principais práticas referidas e nível de evidência

Autores/título	Ano/país/periódico	Método/profissional de saúde	Principais práticas referidas	Nível de evidência
Ribeiro MDA, Castillo IDS, Silva JCA, Oliveira SB “A visão do profissional enfermeiro sobre o tratamento da hanseníase na atenção básica”^(12)^	2017/Brasil/Revista Brasileira em Promoção da Saúde	Estudo descritivo qualitativo, com nove enfermeiros	Consulta de enfermagem; ausência de busca ativa de casos; diagnóstico tardio; tratamento supervisionado; reações adversas ao medicamento; manejo das reações hansênicas; avaliação dermatoneurológica e do grau de IF; vigilância dos contatos; combate ao estigma; educação em saúde.	IV
Pinheiro JDJG, Gomes SCS, Aquino DMCD, Caldas ADJM “Aptidões cognitivas e atitudinais do enfermeiro da atenção básica no controle da hanseníase”^(13)^	2017/Brasil/Revista Baiana de Enfermagem	Estudo descritivo, com 101 enfermeiros	Consulta de enfermagem; ausência de busca ativa de casos; diagnóstico tardio; déficit de informação sobre tratamento supervisionado; ausência de vigilância dos contatos; educação em saúde.	IV
Pereira AJ, Helene LMF, Pedrazini ES, Martins CL, Vieira CSDCA “Atenção básica de saúde e a assistência em hanseníase em serviços de saúde de um município do Estado de São Paulo”^(14)^	2008/Brasil/Revista Brasileira de Enfermagem	Estudo descritivo-exploratório de base epidemiológica, com dez profissionais de saúde (enfermeiros, médicos, auxiliares de enfermagem, assistente social, psicóloga e secretário municipal de saúde)	Consulta de enfermagem; ausência de busca ativa de casos; diagnóstico precoce; notificação; tratamento supervisionado; avaliação dermatoneurológica e do grau de IF; vigilância dos contatos; orientações sobre a BCG; combate ao estigma; avaliação psicológica, oftalmológica, odontológica e de fisioterapia; visita domiciliar; educação em saúde.	IV
Vieira NF, Lanza FM, Lana FCF, Martinez-Riera JR “Avaliação dos atributos da atenção primária à saúde nas ações de controle da hanseníase”^(15)^	2018/Brasil/Revista Enfermagem UERJ	Estudo avaliativo e transversal, envolvendo 251 profissionais da atenção primária (médico, enfermeiro, agente comunitário de saúde e gestores)	Ausência de busca ativa de casos; diagnóstico tardio; tratamento supervisionado; ausência de avaliação do grau de IF; ausência de vigilância dos contatos; ausência de educação em saúde.	IV
Costa IMD, Morais ATND, Nascimento RGD, Lima JPM, Santos MEDMAD, Dias GADS, et al “Conhecimento do fisioterapeuta da atenção primária à saúde sobre a atuação profissional em pacientes com hanseníase”^(16)^	2022/Brasil/Revista Eletrônica Acervo Saúde	Estudo descritivo, transversal, exploratório e qualitativo, com nove fisioterapeutas	Ausência de busca ativa de casos; diagnóstico tardio; avaliação neurológica simplificada incompleta; educação em saúde; orientações inadequadas sobre o autocuidado.	IV
Oliveira CMD,Linhares MSC, Neto FRGX, Mendes IMVP, Kerr LRFS “Conhecimento e práticas dos agentes comunitários de saúde sobre hanseníase em um município hiperendêmico”^(17)^	2018/Brasil/Saúde em Revista	Estudo exploratório-descritivo, sob abordagem quantitativa e qualitativa, com 51 agentes comunitários de saúde	Conhecimento sobre os sinais e sintomas clínicos da hanseníase; busca ativa de casos; tratamento supervisionado; reações adversas ao medicamento; vigilância dos contatos; orientações sobre a BCG; orientação sobre o autocuidado; visita domiciliar; combate ao estigma; educação em saúde.	IV
Lanza FM, Lana FCF “O processo de trabalho em hanseníase: tecnologias e atuação da equipe de saúde da família”^(18)^	2011/Brasil/Texto & Contexto Enfermagem	Pesquisa qualitativa, com 45 profissionais de saúde (médico, enfermeiro, agente comunitário de saúde e gestores)	Consulta de enfermagem; busca ativa de casos; diagnóstico tardio; tratamento supervisionado; reações adversas ao medicamento; manejo das reações hansênicas; avaliação dermatoneurológica e do grau de IF e vigilância dos contatos; visita domiciliar; educação em saúde.	IV
Rodrigues FF, Calou CGP, Leandro TA, Antezana FJ, Pinheiro AKB, Silva VMD, et al “Conhecimento e prática dos enfermeiros sobre hanseníase: ações de controle e eliminação”^(19)^	2015/Brasil/Revista Brasileira de Enfermagem	Estudo avaliativo com abordagem qualitativa, com 16 enfermeiros	Consulta de enfermagem; busca ativa de novos casos; diagnóstico precoce; tratamento supervisionado; manejo das reações hansênicas; avaliação dermatoneurológica e do grau de IF; vigilância dos contatos; orientações sobre a BCG; combate ao estigma; orientação sobre o autocuidado; visita domiciliar; educação em saúde.	IV
Carvalho APM, Fabri ACOC, Lanza FM, Lopes FN, Lana FCF “Integração das ações de controle da hanseníase sob a perspectiva dos profissionais da saúde”^(20)^	2015/Brasil/Revista Enfermagem UFPE	Estudo qualitativo, com 54 gestores e profissionais de saúde (médico, enfermeiro, técnico de enfermagem, auxiliar de enfermagem e agente comunitário de saúde)	Ausência de busca ativa; diagnóstico tardio; tratamento supervisionado; reações adversas ao medicamento; ausência de avaliação do grau de IF; vigilância dos contatos; visita domiciliar; ausência de educação em saúde.	IV
Sousa GS, Silva RLF, Xavier MB “Atributos da atenção primária em saúde no controle da hanseníase: ótica do enfermeiro”^(21)^	2017/Brasil/Revista Baiana Enfermagem	Estudo de avaliação, com 11 enfermeiros	Consulta de enfermagem; Ausência de busca ativa; diagnóstico precoce; ausência de avaliação dermatoneurológica e do grau de IF; vigilância dos contatos; orientação familiar; educação em saúde.	IV
Oliveira AG, Camargo CG “Hanseníase: conhecimentos teóricos e práticos de profissionais de enfermagem que atuam na atenção básica”^(22)^	2020/Brasil/Salusvita	Estudo de delineamento observacional, transversal e analítico, com 42 enfermeiros	Consulta de enfermagem; ausência de busca ativa; diagnóstico tardio; deficiência na avaliação dermatoneurológica e do grau de IF; déficit de informação sobre tratamento supervisionado.	IV

*BCG - Bacilo Calmette-Guérin; IF – incapacidade física.*

O conteúdo dos estudos foi analisado, e os temas identificados permitiram seu agrupamento conforme similaridade das informações, originando três categorias temáticas: Diagnóstico precoce e tratamento oportuno; Prevenção das incapacidades físicas; e Vigilância dos contatos domiciliares e sociais.

## DISCUSSÃO

É de fundamental importância que os profissionais de saúde, diante dos casos suspeitos ou confirmados de hanseníase, desempenhem sua prática conforme ações preconizadas pelo Programa Nacional de Controle e Eliminação da Hanseníase (PNCEH). A adoção dessas ações e estratégias, com base em um arcabouço teórico-prático recomendado por autoridades em saúde, permite o manejo adequado dos casos, diagnóstico precoce, acesso ao tratamento em tempo oportuno, prevenção das IF e vigilância dos contatos domiciliares, o que garante a recuperação da pessoa atingida pela doença^(23)^.

Nessa perspectiva, utilizaram-se essas ações preconizadas pelo PNCEH para guiar o processo de discussão dos achados identificados nos estudos.

### Diagnóstico precoce e tratamento oportuno

A equipe multiprofissional de saúde se configura capital humano essencial para a valorização das práticas de atenção à saúde na comunidade, desempenhando um trabalho complexo com ênfase na articulação e desenvolvimento do cuidado integral ao usuário^(24)^. O enfermeiro, como um dos profissionais integrantes da equipe, torna-se elemento-chave na execução das ações propostas pelo PNCEH, atuando no cuidado integral dos pacientes, desde o diagnóstico até a conclusão do tratamento^(25)^.

A consulta de enfermagem é de extrema importância nas ações de controle da hanseníase, pois, além de criar confiança e vínculo com o paciente, proporciona informações e esclarecimentos sobre a doença para compreender as manifestações clínicas, a necessidade de adesão ao tratamento, o controle dos comunicantes, a prioridade da cura e a prevenção de incapacidades. Dessa forma, o profissional enfermeiro deve atuar diante das necessidades individuais de cada paciente e da prestação de uma assistência de qualidade^(12, 13, 14, 18, 19, 21, 22)^.

A busca de pacientes suspeitos em hanseníase pode ser realizada de forma ativa e passiva. A busca ativa faz parte do trabalho da equipe da APS por meio do rastreio dos usuários mediante campanhas, exame de coletividade e mobilização da comunidade^(18, 19)^. É uma estratégia fundamental para descoberta de casos da doença que, quando desvalorizada, contribui para ampliar as margens do silêncio epidemiológico^(12, 13, 14, 15, 16, 20, 21, 22)^. A busca passiva dá-se quando os casos procuram a unidade de saúde via demanda espontânea ou encaminhamento, apresentando queixas dermatológicas^(26)^. Essas estratégias, principalmente a primeira, contribuem com o diagnóstico em tempo oportuno, essencial para prevenção de incapacidades e/ou agravo dos casos.

O diagnóstico da hanseníase é clínico-epidemiológico, realizado por meio do exame físico do paciente, no qual observam-se nervos periféricos espessados e/ou lesões de pele ou áreas de pele com alterações de sensibilidade térmica e/ou dolorosa e/ou tátil e alterações autonômicas^(27)^. O diagnóstico precoce e o tratamento adequado são condições essenciais para interromper a transmissão e reduzir as consequências físicas e sociais da doença. Observa-se a ênfase quanto ao diagnóstico em três estudos^(14, 19, 21)^. O diagnóstico tardio parece estar associado à falta de capacitação dos profissionais e à demora no início do tratamento, o que contribui para maior dano neural ao paciente e para o agravamento da doença^(12, 13, 15, 16, 18, 20, 21, 22)^. Destaca-se a ausência da prática do diagnóstico precoce em menores de 15 anos, o que contribui para as taxas relevantes de endemicidade^(19)^.

Após o diagnóstico, inicia-se o tratamento por meio da administração da PQT-U, tratamento preconizado pela Organização Mundial da Saúde (OMS) a partir de 01 de julho de 2021. O tratamento se baseia na combinação de três medicamentos (rifampicina, dapsona e clofazimina) por um período de seis meses para as formas clínicas paucibacilares e 12 meses para as multibacilares^(28)^. As medicações que compõem a PQT-U causam efeitos adversos incômodos, sendo um dos principais motivos para a interrupção ou abandono do tratamento^(29)^. Entre os estudos, destacou-se o déficit de informação dos profissionais sobre o tratamento adequado da hanseníase^(13, 14, 15, 16, 17, 18, 19, 20, 21, 22)^.

A PQT-U possui doses supervisionadas, considerada quando o paciente comparece, a cada 28 dias, ao serviço de saúde para receber rifampicina, dapsona e clofazimina. A ausência do paciente para receber a dose significa a continuidade da transmissão da doença e a resistência do bacilo à medicação^(28)^. Logo, o tratamento supervisionado contribui na redução do abandono do tratamento e aumento do número de pessoas curadas^(12, 14, 15, 17, 18, 19, 20)^.

A adesão ao tratamento é um dos maiores e mais complexos desafios para o sistema de saúde. É importante a visita domiciliar para a construção do vínculo entre o profissional e o paciente^(30)^, para se estabelecer e pactuar as responsabilidades inerentes a cada um desses atores durante o tratamento e possibilitar a atenção integral às demandas do paciente com o objetivo de fortalecer essa relação e desenvolver um plano de cuidados direcionado, considerando que o tratamento da hanseníase demanda um período prolongado^(14, 17, 18, 19, 20)^. Nesse momento, deve-se reforçar sobre a importância da adesão ao tratamento, estimular o autocuidado, fornecer orientações ao paciente e familiares e avaliar os contatos com o intuito de interromper a cadeia de transmissão^(31)^.

Durante o tratamento, podem aparecer intercorrências clínicas, reações adversas ao tratamento^(12, 17, 18, 20)^, reações hansênicas, dúvidas no diagnóstico e conduta. Esses casos impõem a necessidade de encaminhamentos aos serviços de referência^(32)^. As reações hansênicas são fenômenos de aumento da atividade da doença, com piora clínica que podem ocorrer antes, durante ou após o final do tratamento com a PQT-U. Essas reações resultam da inflamação aguda causada pela atuação do sistema imunológico do hospedeiro que ataca o bacilo^(12, 18, 19)^. É recomendável o exame odontológico nos pacientes, pois focos infecciosos podem desencadear o aparecimento de episódios reacionais^(33)^.

A fim de proporcionar uma assistência completa, ressalta-se a importância da colaboração interdisciplinar, da comunicação eficaz e do compromisso contínuo entre os diferentes profissionais que compõem as equipes de saúde, incluindo enfermeiros, dermatologistas, oftalmologistas, psicólogos e terapeutas ocupacionais, com o intuito de favorecer um atendimento integral e de qualidade aos pacientes com hanseníase^(12, 14)^.

### Prevenção das incapacidades físicas

Um dos focos da Estratégia Global da hanseníase 2021-2030, lançada pela OMS em 2021, são as IF, com recomendação de modificações operacionais estratégicas. Entre as novas metas, tem-se zero hanseníase, zero infecção e doença e zero incapacidade até 2030. Essa iniciativa ocorre em resposta ao número crescente de casos com grau 2 de IF que vem ocorrendo na contramão da redução da detecção global da doença^(34)^.

O Grau de Incapacidade Física (GIF) dos pacientes deve ser avaliado no momento do diagnóstico, bem como na alta por cura. Entretanto, essa ação, que é fundamental no manejo da hanseníase, muitas vezes é negligenciada. Entre os estudos analisados, quatro abordaram essa prática^(12, 14, 18, 19)^. A avaliação do GIF é um indicador epidemiológico que determina a precocidade do diagnóstico e o sucesso das atividades com objetivo de interromper a cadeia de transmissão^(28)^.

A avaliação do GIF também possibilita o planejamento de ações de prevenção de incapacidades e a mensuração da qualidade da assistência em saúde^(35)^. É importante que todos os profissionais de saúde estejam devidamente capacitados para realizar a avaliação do GIF em pacientes com hanseníase. Contudo, os resultados da pesquisa apontam para uma lacuna preocupante nessa competência entre os profissionais que atuam na APS. No contexto analisado, identificaram-se deficiências no que diz respeito à avaliação neurológica simplificada (ANS) e à capacidade de realizar testes de força muscular e sensibilidade dos olhos, mãos e pés, o que sugere a necessidade de melhor preparo desses profissionais^(13, 15, 16, 20, 21, 22)^.

A ANS é preconizada pelo Ministério da Saúde como meio de avaliar os comprometimentos provenientes das lesões nervosas causadas pelo M. *leprae.* A avaliação do GIF é uma medida que indica a existência de perda da sensibilidade protetora e/ou deformidade visível em consequência de lesão neural, direcionando o profissional para práticas de prevenção de incapacidades e reabilitação. Essa avaliação identifica as deficiências sensório-motoras nos olhos, mãos e pés, e as classifica em GIF 0, 1 e 2. O grau 0 caracteriza o paciente que não manifesta qualquer problema causado pela hanseníase nas mãos, pés e olhos; o grau 1 ocorre quando a IF não é detectável pela inspeção ou pelo teste de acuidade visual, mas observa-se diminuição da sensibilidade protetora ou redução da força muscular nas mãos, pés e/ou olhos; e o grau 2 é determinado pela presença de deficiências físicas visíveis ou perda da acuidade visual ocasionada pela neuropatia hansênica^(1)^.

O tropismo da bactéria por nervos periféricos é o responsável pelo potencial incapacitante da doença, que pode gerar deformidades e incapacidades, em especial nos olhos, mãos e pés. É de suma importância ressaltar que o tratamento e a gestão da hanseníase requerem uma abordagem interdisciplinar, além da colaboração ativa entre o profissional de saúde e o paciente, por meio do diagnóstico precoce e acompanhamento desde o início até a alta, para prevenir ou minimizar as IF que causam limitações e promovem preconceito e exclusão social nos acometidos^(36)^.

### Vigilância dos contatos domiciliares e sociais

A vigilância dos contatos domiciliares é outra das ações importantes no controle da hanseníase na APS por meio da detecção ativa de casos, considerando que eles representam uma população com maior risco de adoecimento devido à maior exposição ao bacilo. Deve-se incluir, na anamnese em relação aos sinais e sintomas da doença, exame dermatoneurológico de todos os contatos dos casos novos, independentemente da classificação operacional, imunoprofilaxia com vacina Bacilo Calmette-Guérin (BCG), bem como seguimento por um período de pelo menos cinco anos após o diagnóstico do caso notificado^(28)^.

A vigilância de contatos visa à descoberta de casos novos entre aqueles que convivem ou conviveram, de forma prolongada, com o caso novo de hanseníase diagnosticado^(12, 14, 17, 18, 19, 20, 21)^, além de descobrir possíveis fontes de infecção no domicílio (familiar) ou fora dele (social)^(37)^. Os desafios para o controle da hanseníase incluem a continuidade da transmissão do bacilo, dificuldades na vigilância de contatos e conhecimento restrito sobre a transmissão. A prevenção da hanseníase baseia-se na detecção dos contatos dos pacientes^(38)^, em especial contatos familiares, uma vez que é o principal determinante para a permanência dos níveis de incidência^(20)^. É utilizada a avaliação dermatoneurológica para avaliar a presença de sinais de hanseníase em contato.

A avaliação dermatoneurológica, peça-chave no diagnóstico da hanseníase, não tem sido realizada com a frequência esperada na avaliação dos contatos. Deve ser realizada anualmente, durante cinco anos, independentemente se são familiares ou não, pois são os mais vulneráveis a desenvolver a doença^(39)^. Essa prática, quando não realizada, contribui para a propagação do bacilo e desenvolvimento de IF, pois muitos profissionais não se sentem seguros ou capacitados o suficiente para realizá-la nos contatos^(15, 16, 20, 21)^. Na primeira avaliação, caso o contato não apresente sinais de hanseníase, deve ser avaliado sobre a situação vacinal da BCG e encaminhado à sala de vacina se necessário.

A vacina BCG deve ser aplicada nos contatos sem presença de sinais e sintomas da doença no momento da avaliação dermatoneurológica^(14, 17, 18, 19)^. A administração da vacina depende do histórico vacinal e/ou presença de cicatriz vacinal, e segue a seguinte orientação: ausência de cicatriz vacinal de BCG- uma dose; uma cicatriz vacinal – uma dose; duas cicatrizes vacinais – não vacinar^(37)^. Na maioria dos estudos revisados^(12, 13, 15, 16, 18, 20, 21, 22)^, não foram observados orientação e encaminhamento dos contatos para a realização da BCG. A falta de orientações no momento das consultas pode ser minimizada com a prática da educação popular em saúde que, desenvolvida por todos os protagonistas do cuidado em saúde, torna-se uma ferramenta de grande impacto.

A educação em saúde em relação à hanseníase é uma ferramenta de interação das práticas de saberes envolvidos no processo de saúde-doença^(12, 14, 17, 18, 19, 21)^ capaz de potencializar processos que implicam diagnóstico precoce, conscientização, prevenção da transmissão, adesão ao tratamento, promoção da saúde mental, prevenção de incapacidades e apoio comunitário. A enfermagem exerce papel nuclear no processo de capacitação para a prevenção e promoção do cuidado desse paciente^(40)^. Essa prática, quando não realizada por falta de conhecimento do profissional em relação à doença, pode contribuir para o diagnóstico tardio e não adesão ao tratamento^(13, 15, 16, 20, 22)^.

### Limitações do estudo

Uma das limitações identificadas está relacionada ao fato de a revisão ter sido processada apenas com produções nacional, o que limita a comparação com a realidade de outros países e regiões do mundo.

### Contribuições para a área da saúde

A identificação das evidências permitiu apresentar uma síntese das práticas adotadas, contribuindo com a divulgação do conhecimento entre a comunidade, profissionais e serviços de saúde, com o intuito de melhorar a atenção relacionada ao controle dos casos de hanseníase.

## CONSIDERAÇÕES FINAIS

O estudo permitiu identificar as principais práticas adotadas pelos profissionais de saúde da APS em relação à hanseníase, como manejo das reações hansênicas, avaliação dos contatos, combate ao estigma, educação em saúde, tratamento supervisionado, visita domiciliar, orientação sobre o autocuidado, busca de novos casos, entre outras. Contudo, tornou-se preocupante um achado que aponta para a negligência de ações prioritárias ao controle da hanseníase, tais como o diagnóstico precoce, realização da ANS e de atividades relacionadas à vigilância dos contatos domiciliares e sociais.

Desse modo, observa-se a necessidade de alinhamento das práticas realizadas com o preconizado pelo PNCEH, tendo em vista que algumas não foram identificadas nos estudos revisados. Na APS, é de grande importância a implementação de iniciativas destinadas à educação continuada dos profissionais de saúde com o propósito de fortalecer, por exemplo, a capacitação técnica desses profissionais, visando aprimorar a eficácia no controle e eliminação da hanseníase como problema de saúde pública.
